# THE POTENTIAL BENEFITS OF COMPANION ROBOTIC PETS ON THE QUALITY OF LIFE AMONG COMMUNITY-DWELLING OLDER WOMEN

**DOI:** 10.1093/geroni/igad104.2881

**Published:** 2023-12-21

**Authors:** Suk-Young Kang, Jeungkun Kim, Antonina Poplawski

**Affiliations:** Binghamton University, State University of New York, Binghamton, New York, United States; Kangnam University, Yongin, Republic of Korea; Binghamton University, State University of New York, Binghamton, New York, United States

## Abstract

This project explores whether companion robotic pets improve mental well-being of community-dwelling older women with depression. Depression is a major mental health issue disproportionately impacting women due to lifelong experiences of sexism and limitations in education and employment. Aging exacerbates an already undervalued status, and feelings of powerlessness increase. Women thus experience double jeopardy from discrimination on both gender and age (Hooyman et al., 2017). This project will investigate the efficacy of companion robotic pets and will recommend plans to the local Office for Aging (OFA). Recruitment was done in collaboration with the local OFA, with mail-survey data collected from community-dwelling women over the age of 65. 38 participants were provided with a robotic pet after an initial screening. Following a one-group pretest posttest design, data from 31 participants were used to determine the impact of a robotic pet intervention on older women’s quality of life. Paired samples t-tests were used to compare means of the 15-item Geriatric Depression Scale (GDS), 10-item Geriatric Anxiety Scale (GAS), 6-item De Jong Loneliness Scale, and one physical health status question completed by 30 women. There was a significant average difference between before- and after-companion pet scores for depression (t29 = 6.597, p < 0.001), anxiety (t29 = 6.728, p < 0.001), loneliness (t29 = 6.462, p < 0.001) and physical health (t29 = - 3.496, p = 0.002). Results confirmed companion robotic pets improve mental health in older women with depression and anxiety. Moreover, they are helpful in improving loneliness and physical health.

